# Electrical detection and quantification of single and mixed DNA nucleotides in suspension

**DOI:** 10.1038/srep34016

**Published:** 2016-09-28

**Authors:** Mahmoud Al Ahmad, Neena G. Panicker, Tahir A. Rizvi, Farah Mustafa

**Affiliations:** 1 Department of Electrical Engineering, College of Engineering, United Arab Emirates University, Al Ain, UAE; 2 Zayed Bin Sultan Center for Health Sciences Division United Arab Emirates University, Al Ain, UAE; 3 Department of Biochemistry, College of Medicine and Health Sciences, United Arab Emirates University, Al Ain, UAE; 4 Department of Microbiology and Immunology, College of Medicine and Health Sciences United Arab Emirates University, Al Ain, UAE

## Abstract

High speed sequential identification of the building blocks of DNA, (deoxyribonucleotides or nucleotides for short) without labeling or processing in long reads of DNA is the need of the hour. This can be accomplished through exploiting their unique electrical properties. In this study, the four different types of nucleotides that constitute a DNA molecule were suspended in a buffer followed by performing several types of electrical measurements. These electrical parameters were then used to quantify the suspended DNA nucleotides. Thus, we present a purely electrical counting scheme based on the semiconductor theory that allows one to determine the number of nucleotides in a solution by measuring their capacitance-voltage dependency. The nucleotide count was observed to be similar to the multiplication of the corresponding dopant concentration and debye volume after de-embedding the buffer contribution. The presented approach allows for a fast and label-free quantification of single and mixed nucleotides in a solution.

Quick, label-free screening and quantification of DNA bases is becoming increasingly important^1^,^2^,^3^. The existing DNA sequencing techniques need extensive sample preparation which make them costly and time consuming. Several methods have been introduced to investigate the processing, manipulation, and characterization of single DNA molecules^1^,^2^,^3^,^4^,^5^,^6^,^7^,^8^,^9^,^10^,^11^,^12^,^13^,^14^,^15^,^16^,^17^. A suggested alternative for establishing a quick and reliable label-free method is to use electrical-based techniques^4^. The conventional DNA sequencing technologies that are being used to identify nucleotides commonly utilize light emission^5^. This process includes a polymerase chain reaction (PCR) step for the amplification of template sequences which helps to produce sufficient material to generate detectable corresponding signals^6^. An ideal DNA sequencing technology should have the ability to detect single nucleotides directly by observing changes in a corresponding electric parameter, thus eliminating the need for both fluorescent probes and PCR amplification. To achieve this, different innovative methods are being tried. For example, nano pores are being used within which each nucleotide in a DNA strand can translocate through^7^,^8^,^9^. This specifically modulates the ionic current being passed through the nano device which allows identification of each nucleotide, and eventually the sequence of the entire stretch of DNA^10^,^11^. In another approach, though controversial^18^,^19^, DNA sequences are being identified via tunneling currents conducted as the sequences pass between a pair of nano electrodes^12^. Furthermore, numerous studies have shown the ability of charge transfer in DNA through quantum tunneling with potential applications^13^,^14^,^15^,^16^,^17^. Nevertheless, a quick and rapid systematic label-free DNA quantification and identification technique incorporating low noise interference by electrical parameters has not yet materialized.

This work investigates the electrical properties of single and mixed nucleotides in solution. The four types of nucleotides tested represent the basic building blocks in double strands of DNA with their own unique structures (Fig. 1). Nucleotides are composed of a five-carbon (pentose) sugar molecule attached to a phosphate group (Fig. 1). A free hydroxyl at position 1 of the deoxyribose sugar is used to tether four different types of nitrogenous bases, adenine, guanine, cytosine, and thymine, leading to the formation of the purine nucleotides (dexoyadenosine and deoxyguanine triphosphates, dATP and dGTP, respectively) and the pyrimidine nucleotides (deoxycytosine and deoxythymine, dCTP and dTTP, respectively) (Fig. 1). As can be seen, the two groups of nucleotides differ in their structures with the purines being the larger of the two, containing a two-ringed structure, while the pyrimidines containing a single-ringed structure. Furthermore, differential modifications of the ringed structures give each nucleotide its unique chemical structure. Finally, it is the number and sequence of the incorporated nucleotides that impart a distinct identity to each molecule of DNA.

To examine the electrical properties of single and mixed nucleotides for their detection and quantification, capacitance-voltage measurements were performed on nucleotides in solution. Nucleotides are randomly distributed at the molecular level within a suspension. When these suspended nucleotides are subjected to an electric field, they get polarized to different degrees depending upon their molecular structures. The strength of this polarization depends upon the ability of the nucleotides to hold electrical charges as well as their interaction with the medium^10^. To detect DNA nucleotides suspended in a solution electrically, the nucleotide suspension (buffer plus nucleotides) and the corresponding buffer solution (nucleotide-free solution) were loaded inside a capacitive structure to perform capacitance-voltage measurements for each nucleotide solution. The capacitance contribution of the control buffer was subtracted from that of the samples since the buffer can act as a linear superposition to that of sample or, equivalently, the capacitance value of the control can be parallel to that of the sample. This “parallel model” was based on our observations that the effective capacitance of the nucleotide suspension (nucleotide + control buffer) was higher than the capacitance of the nucleotide-free control medium. If the effective capacitance of the suspension was lower than the reference, then a “series model” would have been considered.

## Results and Discussion

### Electrical Detection of Nucleotides

**Figure 2:**
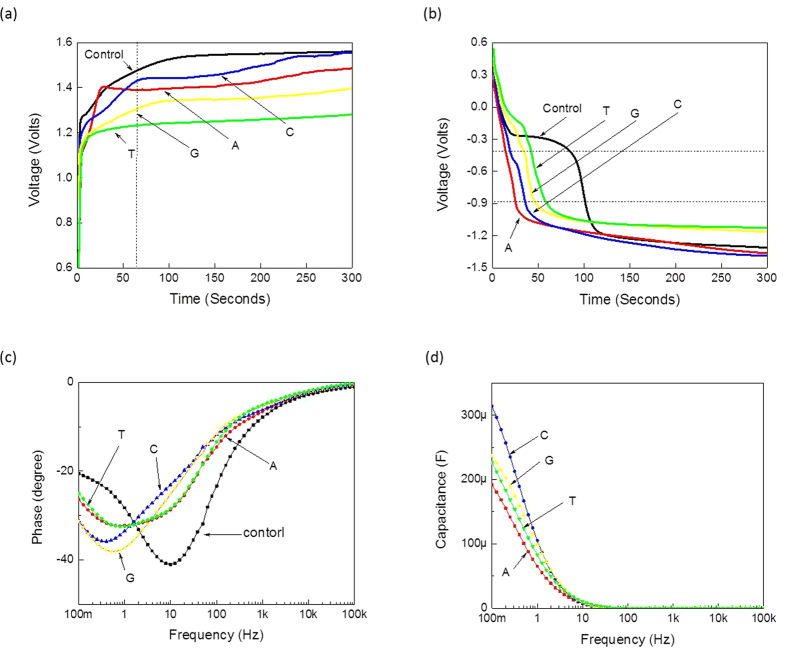
Characterization of the electrical properties of DNA nucleotides: (**a**) charging (**b**) discharging profiles, (**c**) phase profiles versus frequency, and (**d**) extracted capacitances of DNA nucleotides. A 1:100 dilution of the nucleotides was tested from a stock solution of 100 Mm, resulting in a final concentration of 1 mM. A, C, G and T are dATP, dCTP, dGTP and dTTP, respectively. Control = Tris-EDTA buffer without nucleotides.

To detect the individual nucleotides electrically and extract relevant parameters, the four nucleotides were diluted individually in control TE buffer (10 mM Tris-Cl, 1 mM EDTA, pH 8) and loaded inside an open-ended coaxial cable^20^ of Gamry 3000 instrument (USA)^21^ that has a wide range of electrical measurement capabilities. One way to demonstrate the electrical polarization of DNA nucleotides is through the electrical charging-discharging measurements. Therefore, tests of the electrical charging and discharging pofiles for the nucleotide suspensions were conducted and are shown in Fig. 2. The measured potential (voltage) in both profiles (Fig. 2(a,b)) is the potential value that builds between the two electrodes as a constant current of 0.5 mA flows between them. These profiles were different when compared with the control TE buffer which was used to suspend the nucleotides (Fig. 2(a,b)). The differences reveal a direct demonstration of the polarization capabilities of the four nucleotides of DNA, dATP, dCTP, dGTP and dTTP, when subjected to an electrical field. For the sake of simplicity, these four nucleotides will be referred to as A, C, G and T, respectively, for the remainder of the manuscript.

As can be seen from Fig. 2(a), it was easy to observe differences between the different nucleotides and the control medium within the time period from ~65 to 300 seconds, a region highlighted by dashed vertical lines. The charging of DNA nucleotide suspensions was instantaneous and presence of the individual nucleotides increased the charging time of the control buffer. The number of molecules in each nucleotide suspension was calculated to be 6.022 × 10^17^ molecules in 1 milliliter stock of TE buffer. To compute the charging rate values, the contribution of the control buffer was first de-embedded by subtracting it from the individual nucleotide profiles. These values (charging rates) were computed by fitting each charging profile, between 65 and 300 seconds, with the linear formula: Y = αX + β, where Y is the charging profile, X is the time step, β is the y intercept and α is the slope of the profile. The corresponding slope values represent the charging rates of the individual nucleotides. Based on curve fitting, we observed that the charging rate of C, A, G and T in their respective suspensions was 0.63, 0.51, 0.28 and 0.09 mVolts per second. To extract the charging rate per single DNA nucleotide, the computed charging rate was then divided over the total number of nucleotides in each suspension. Therefore, the charging rate per one molecule of C, A, G and T was calculated to be 1.04, 0.84, 0.46 and 0.15 zepto Volts per second, respectively.

The relative charging rate of the four nucleotides revealed that C showed the highest rate, followed by A, and G, while T showed the lowest value; thus all four nucleotides showed differences in their charging rates. This is despite the fact that the nucleotide suspensions were prepared with equal amounts of the four different nucleotides in the same buffer and volume and the same amounts of the different suspensions were measured in the coaxial adaptor at the same time interval with the application of identical electrical current applications. Since the only difference between the four tested nucleotide samples was in their biochemical composition, we believe that their composition and structure is responsible for the pronounced differences observed in their electrical properties and characteristics (Fig. 2).

This assertion can be supported by the following observation: if we consider the charging rate per one molecule of C, A, G and T (which was calculated to be 1.04, 0.84, 0.46 and 0.15 zepto Volts per second, respectively), the sum of the charging rates of the corresponding complementary DNA base pairs AT and CG becomes 0.99 and 1.5 zepto Volts per second, respectively. Thus, the ratio between the sum of charging rates between CG and AT is 1.5/0.99, which is around 1.5. Now it is well known that there are two covalent bonds between A and T and three between C and G; thus, the capacitance between C and G is 1.5 times higher than that for A and T, reflecting the same ratio observed in their actual charging rates.

The discharging profiles of the nucleotides further provided specifics about the physics of DNA nucleotides (Fig. 2(b)). By removing the electric field, the nucleotide suspensions returned to their original states within a specific time interval needed to establish the charge equilibrium in the suspension. Discharging occurred due to the redistribution of charges bound within the suspension under the influence of a receding electric field during the discharging process, whereby charges of the opposite polarity and same magnitudes were displaced to different locations within the suspension. At specific discharging voltage range, as represented by the horizontal area between the two dashed lines in Fig. 2(b), the discharging profiles could be distinguished clearly. Each curve exhibited its own falling time, which on average was 19, 29, 38, 47 and 95 seconds for A, C, G, T and control, respectively. This data further shows that the DNA nucleotides reduced the discharging time constant of the control TE buffer.

The charging profiles further revealed that the effective dielectric constants of the nucleotide suspensions were higher than that of the control medium. This was confirmed by the impedance measurements, as demonstrated by Fig. 2(c), which represents phase versus frequency for DNA nucleotides as well as the control medium. As observed, the presence of DNA nucleotides shifted the phase frequency profile towards the lower end when compared to the control nucleotide-free medium (Fig. 2(c)). As concluded from the phase frequency measurements, without DNA nucleotides (buffer alone) the minimum phase was set around 10 Hz. With the addition of DNA nucleotides, the effective dielectric constants of the suspension (DNA nucleotides and media) shifted the minimum phase down in frequency to around less than 1 Hz. The change in minimum phase magnitude indicates that the DNA nucleotides introduced DC resistance which caused a loss in power. Thus, this observation confirms that capacitance measurements can be used to detect the existence of DNA nucleotides inside a buffer media. It was also noticed that the A and T phase profiles were almost identical to each other, whereas C and G exhibited similar phase frequency profiles, despite the fact that A and T have different structures (purine versus a pyrimidine), and the same applies to G and C. In fact, A and T are complimentary bases, while C and G are also complimentary to each other. Next, the capacitance values of each nucleotide were extracted by subtracting the contributions from the control TE buffer since any possible interactions with the buffer could affect the polarization of the nucleotides. As observed in Fig. 2(d), the corresponding capacitances depended upon the nucleotide composition and structure since each nucleotide suspension could still be distinguished from each other.

### Electrical Quantification of Nucleotides

Next, we focused on quantifying the amount of nucleotides suspended in a medium by using the principles of the semiconductor theory^22^,^23^. The basic premise behind the idea was that application of an electric field should lead to the polarization of the biotic nucleotide particles, the strength of which should depend upon the composition of the nucleotides and their concentration. Thus, the nucleotides could be present as an electrical dipole or “impurities” (dopants) that exist in a non-intrinsic semiconductor material (the buffer). One could then calculate the doping concentration of the suspension and de-embed the contribution of the reference medium. Although nucleotides are a miniscule proportion of the surrounding media, they still produce enough change in its electrical properties to be detected, which depends upon the accumulated charge of the nucleotide.

Based on this idea, the dopant concentrations of the nucleotide could be extracted from the de-embedded capacitance-voltage measurements. The number of nucleotides in a suspension (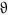) was estimated using Eq. (1):


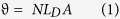


where, *N* is the corresponding doping concentration, *L*_*D*_ is debye electrical length and *A* is the area. Both *N* and *L*_*D*_ were determined from the capacitance–voltage measurements after de-embedding the buffer control contribution, as given in Eqs (2) and (3), respectively^21^:


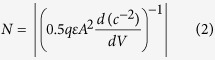



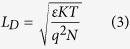


where, *q* is the electron charge, *ε* is the dielectric constant of the nucleotide suspension (or medium), *A* is the overlapping area of the effective capacitance, *C* is the measured capacitance, *V* is the applied voltage. *K* is Boltzmann constant 1.38 × 10^−23^ J/K, and *T* is the temperature in kelvin.

To electrically quantify and determine whether the individual nucleotides could be counted in mixed nucleotide solutions, a full fractional experiment was conducted, as illustrated in Table 1. As before, the resulting single and mixed nucleotide suspensions were prepared and loaded into the open-ended coaxial cable and data gathering was achieved by using Gamry 3000.

**Figure 3:**
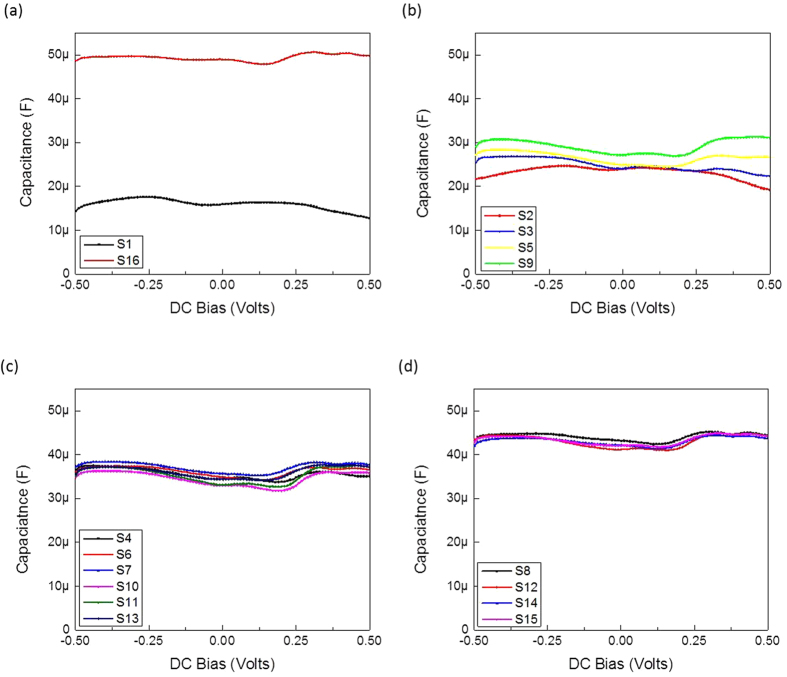
Capacitance versus voltage measurements for the mixing experiment illustrated in Table 1 conducted at 5 Hz. (**a**) Capacitance versus DC bias for DNA free suspension (S1) and the quadruple mixed combination ACGT (S16); (**b**) Capacitance versus DC bias for all possible single DNA nucleotides A, C, G and T (S2, S3, S5 and S9, respectively); (**c**) Capacitance versus DC bias for all possible double combinations AC, AG, AT, CG, CT and GT (S4, S6, S7, S10, S11 and S13, respectively). (**d**) Capacitance versus DC bias for all possible triple combinations ACG, ACT, AGT and CGT (S8, S12, S14 and S15, respectively). A, C, G and T are dATP, dCTP, dGTP and dTTP, respectively.

Results of the corresponding samples for their capacitance versus voltage profiles are shown in Fig. 3. The TE control buffer (sample S1) showed the lowest capacitance value, which increased to the maximum in the presence of the four nucleotides (sample S16) (Fig. 3(a)). When the TE buffer was supplemented with each single nucleotide individually, subtle difference between the capacitance versus voltage profiles of individual nucleotides could be observed (samples S2, S3, S5 and S9) (Fig. 3(b)).

This difference disappeared when double and triple nucleotide mixes were tested (Fig. 3(c,d)), revealing identical profiles. These results suggest that the capacitances versus voltage profiles are a characteristic primarily of the dose of the nucleotides that can be used for identification and sequencing purposes.

**Figure 4:**
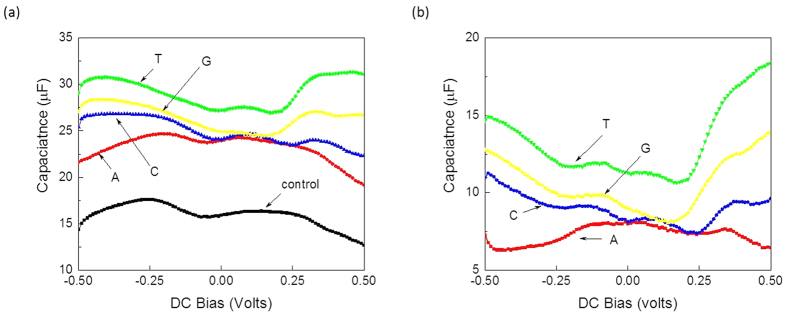
Related electrical measurements of the individual DNA nucleotides: (**a**) capacitance-voltage measurements before de-embedding, and (**b**) capacitance-voltage measurements after de-embedding effects of the buffer. A, C, G and T are dATP, dCTP, dGTP and dTTP, respectively.

Figure 4 highlights the capacitances of nucleotides before and after de-embedding the contribution of the control buffer. The largest dielectric constant was observed for T, followed by G, C, and A, respectively, followed by the control with the lowest dielectric constant (Fig. 4(a)). By de-embedding the buffer contribution, the corresponding capacitances for the four DNA nucleotides were estimated (Fig. 4(b)). As can be seen clearly, each nucleotide exhibited its own capacitance profile, even after subtracting the effects of the buffer medium.

### Validation and Sensitivity of the Electrical Approach

After applying the presented theory of electrical parameter extraction algorithm (Equations (1, 2, 3)), a set of corresponding extracted values for the different nucleotides were obtained that are shown in Table 2 along with their actual values as calculated using the known concentrations and Avogadro’s number. The intrinsic dopant concentration, debye length and count for the nucleotide-free buffer are listed in the first row (S1). Comparison of the debye length of the buffer with that of the DNA nucleotides revealed that the length decreased and increased, respectively, depending upon the nature of the DNA nucleotides. As can be seen, the results from these two vastly different techniques (electrical versus calculated) were within one order of magnitude, demonstrating the validity of the proposed electrical technique for nucleotides quantitation.

Furthermore, Table 2 shows that the dopant concentration increased as the number of nucleotide combinations increased. Thus, the quadruple nucleotide mix had the highest dopant concentration, while the single nucleotide mix had the lowest dopant concentration. Also there was a change in the debye length corresponding to the number of DNA molecules per ml. Initially the length assumed its maximum value, but with incremental increase in numbers of nucleotide molecules, this length became shorter as the effective capacitance value increased as shown in Fig. 3.

**Figure 5:**
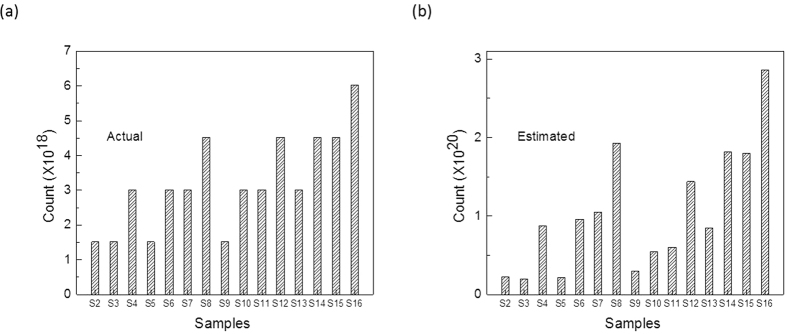
Comparison of the estimated and actual values of nucleotides using (**a**) biochemical and (**b**) electrical techniques. Panel (**a**) shows the actual counts, while panel (**b**) shows the estimated counts of the nucleotides used in the mixing experiment using the electrical technique.

Figure 5 presents a comparison of the actual and estimated nucleotide numbers obtained (shown in Table 2). As can be seen, the overall trend of estimating the nucleotide numbers was remarkably similar with the electrical technique clearly being able to detect nucleotides according to their concentration (no of molecules in solution per volume). Thus, the electrical technique was able to predict the concentration trends even though the actual numbers estimated were off by a magnitude or two, demonstrating the potential of the proposed electrical technique for nucleotides quantification.

**Figure 6:**
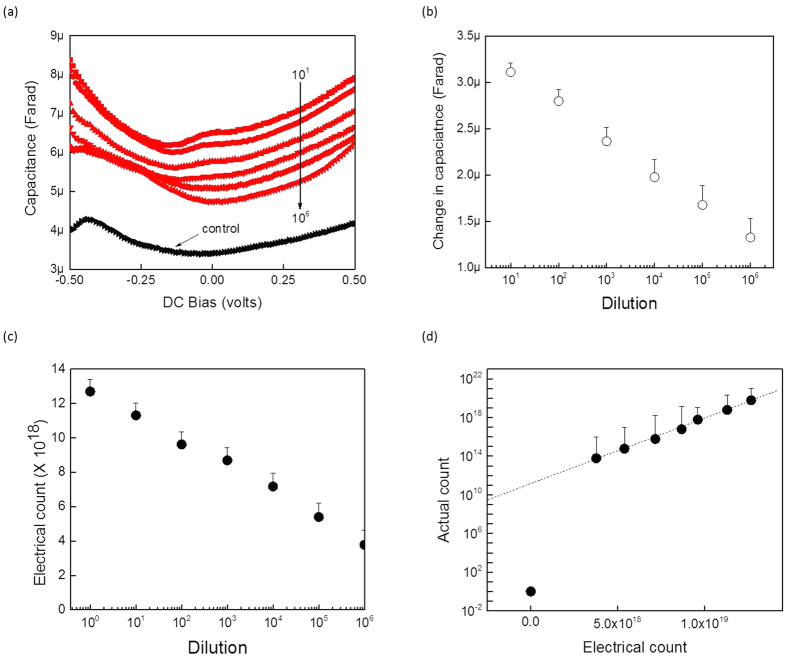
Sensitivity assessment of the electrical method for nucleotide quantitation. The nucleotide dATP was diluted serially from 10^−1^ to 10^−6^ and then characterized electrically. (**a**) Capacitance versus voltage for different dilutions; (**b**) Change in capacitance versus dilution; (**c**) Electrical count versus dilution; and (**d**) Actual count versus electrical count.

The sensitivity of the electrical method was tested by diluting a known concentration of nucleotide dATP with the blank buffer. As expected, the more concentrated the nucleotide suspension, the higher the observed effective capacitance value Fig. 6(a). The change in capacitance as a function of the extracted nucleotide concentration is depicted in Fig. 6(b). As can be observed, the change in capacitance was small, probably due to the proportionally smaller size of nucleotides existing in a much larger volume of buffer. The corresponding extracted electrical counts versus dilutions are shown in Fig. 6(c). As can be seen, the reduction in electrical count was linear and correlated well with the reduction in capacitance observed with their dilution. Due to the comparatively lower volume of the nucleotides in solution compared to the buffer, it is suggested to establish a direct connection between the actual nucleotides with the corresponding extracted electrical count. Figure 6(d) represents such a calibration curve which shows that we could detect nucleotides down to 10^9^ molecules as concluded from the y-intercept of the plotted linear line. This limit of detection is due to the coaxial adaptor structure used in this experiment. For further sensitivity and lower limit of detection, a micro/nano-based capacitance structure should be used.

The precision of the electrical method of measurement depends upon the precision of the capacitance analyzer, which is subject to various sources of noise. The measured CV profiles in this work showed a smooth behavior without ripple levels in all conducted measurements, revealing that high precision could be attained despite using a macro-capacitive structure.

## Conclusions

In summary, our results reveal that deoxyribonucleotides exhibit strong electrical polarization capabilities. This property can be used to detect the different nucleotides electrically employing capacitance-voltage measurements. The electrically-measured and known nucleotide counts correlate well with a limit of detection of 10^9^ molecules, suggesting high sensitivity and simplicity of the present approach. The accurate detection, quantification and sequence analysis of DNA nucleotides in double strands of DNA is one of the most significant challenges of today.

## Materials and Methods

### Nucleotides

The nucleotides tested for these studies were purchased from Fermentas (Thermo-Fischer) or Promega at a concentration of 100 mM. Experiments were conducted using nucleotides at 1 mM concentration. To perform the electrical measurements, individual or a combination of nucleotides were diluted in a buffer containing 10 mM Tris-Cl and 1 mM EDTA, pH 8, (TE buffer).

### Electrical characterization method

Electrical measurements were performed using the Gamry Reference 3000 equipment (Gamry/USA). The measurements of current-voltage, capacitance-voltage, charging, discharging as well as the impedance values were conducted over a frequency range from 10 μHz to 100 kHz. Measurements were performed at an applied oscillation voltage of 15 mVpp. The measurements setup system was calibrated using the manufacture-provided calibration kit. A typical calibration excludes the effect of losses and phase shifts that could add noise to the measured signal from the lengthy used cables.

A two-electrode system was used in this work. The two-electrode is the simplest cell setup but often results in complex results that require more analysis and interpretation when compared to a three-electrode set up. The two-electrode experiment measures the complete voltage dropped by the current across the whole cell. In this work, a coaxial cable adaptor^24^ was used as a two-electrode cell to perform the measurements.

The accuracy and reproducibility of the presented method has been checked using repeated electrical measurements against multiple nucleotides stocks prepared at different times over the same applied bias voltage.

## Additional Information

**How to cite this article**: Ahmad, M. A. *et al.* Electrical detection and quantification of single and mixed DNA nucleotides in suspension. *Sci. Rep.* **6**, 34016; doi: 10.1038/srep34016 (2016).

## Figures and Tables

**Figure 1:**
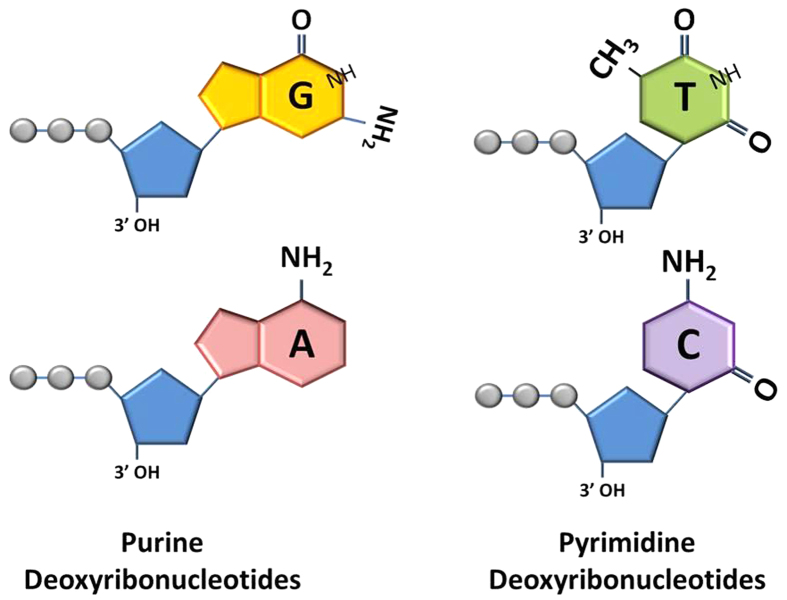
Illustration of the structure and groups of the four types of DNA nucleotides tested.

**Table 1:** Design of the nucleotide mixing experiment*.

Nucleotide/Sample #	S1	S2	S3	S4	S5	S6	S7	S8	S9	S10	S11	S12	S13	S14	S15	S16
dATP	0	1	0	1	0	1	0	1	0	1	0	1	0	1	0	1
dCTP	0	0	1	1	0	0	1	1	0	0	1	1	0	0	1	1
dGTP	0	0	0	0	1	1	1	1	0	0	0	0	1	1	1	1
dTTP	0	0	0	0	0	0	0	0	1	1	1	1	1	1	1	1

^*^0 = Absence of specific nucleotide; 1 = presence of specific nucleotide.

**Table 2:** Electrical parameters for the different types of nucleotides along with their relevant control media.

Sample #	Description	Dopant [m^−3^]	DebyeLength [m]	Estimated Count[No of Nucleotides]	Actual Count[No of Molecules/ml]
S1	Buffer		8.12E-07		NA
S2	A	1.67E + 25	6.70E-07	2.24E + 19	1.51E + 18
S3	C	1.35E + 25	7.52E-07	2.02E + 19	1.51E + 18
S4	AC	1.10E + 26	3.99E-07	8.76E + 19	3.01E + 18
S5	G	1.36E + 25	7.87E-07	2.14E + 19	1.51E + 18
S6	AG	1.29E + 26	3.71E-07	9.58E + 19	3.01E + 18
S7	CG	1.50E + 26	3.52E-07	1.05E + 20	3.01E + 18
S8	ACG	3.64E + 26	2.65E-07	1.93E + 20	4.52E + 18
S9	T	2.06E + 25	7.16E-07	2.96E + 19	1.51E + 18
S10	AT	4.65E + 25	5.85E-07	5.44E + 19	3.01E + 18
S11	CT	5.65E + 25	5.34E-07	6.02E + 19	3.01E + 18
S12	ACT	2.19E + 26	3.29E-07	1.44E + 20	4.52E + 18
S13	GT	1.04E + 26	4.08E-07	8.46E + 19	3.01E + 18
S14	AGT	3.37E + 26	2.70E-07	1.82E + 20	4.52E + 18
S15	CGT	3.32E + 26	2.72E-07	1.80E + 20	4.52E + 18
S16	ACGT	6.57E + 26	2.17E-07	2.86E + 20	6.02E + 18

## References

[b1] Star A. *et al.* Label-free detection of DNA hybridization using carbon nanotube network field-effect transistors. J. Proc Natl Acad Sci. 103, 921–926 (2006). 10.1073/pnas.0504146103 PMC1347966 16418278

[b2] Gao Z. *et al.* Silicon nanowire arrays for label-free detection of DNA. J. Anal Chem. 79, 3291–3297 (2007). 10.1021/ac061808q 17407259

[b3] Liu S., Li C., Cheng J. & Zhou Y. Selective photoelectrochemical detection of DNA with high-affinity metallointercalator and tin oxide nanoparticle electrode. J. Anal Chem. 78, 4722–4726 (2006). 10.1021/ac052022f 16808488

[b4] Huang S. *et al.* Identifying single bases in a DNA oligomer with electron tunnelling. Nat Nanotechnology 5, 868–873 (2010). 10.1038/nnano.2010.213 PMC4121130 21076404

[b5] Shokralla S., Spall J. L., Gibson J. F. & Hajibabaei M. Next-generation sequencing technologies for environmental DNA research. J. Mol Ecol. 21, 1794–1805 (2012). 10.1111/j.1365-294X.2012.05538.x 22486820

[b6] Kalle E., Kubista M. & Rensing C. Multi-template polymerase chain reaction. J. Biomolecular Detection and Quantification. 2, 11–29 (2014). 10.1016/j.bdq.2014.11.002 PMC5121205 27896140

[b7] Branton D. *et al.* The potential and challenges of nanopore sequencing. J. Nat Biotechnol. 26, 1146–53 (2008). 10.1038/nbt.1495 PMC2683588 18846088

[b8] Zwolak M. & DiVentra M. Physical approaches to DNA sequencing and detection. J. Rev Mod Phys. 80, 141–165 (2008).

[b9] Clarke J. *et al.* Continuous base identification for single-molecule nanopore DNA sequencing. J. Nat Nanotechnol. 4, 265–270 (2009). 10.1038/nnano.2009.12 19350039

[b10] Stoddart D. *et al.* Single-nucleotide discrimination in immobilized DNA oligonucleotides with a biological nanopore. J. Proc Natl Acad Sci. 106, 7702–7707(2009). 10.1073/pnas.0901054106 PMC2683137 19380741

[b11] Zwolak M. & DiVentra M. Electronic signature of DNA nucleotides via transverse transport. J. Nano Lett. 5, 421–424 (2005). 10.1021/nl048289w 15755087

[b12] Ohshiro T. *et al.* Single-molecule electrical random resequencing of DNA and RNA. J. Sci Rep. 2, 501 (2012). 10.1038/srep00501 PMC3392642 22787559

[b13] Albrecht T. Electrochemical tunnelling sensors and their potential applications. J. Nat Commun. 3, 829 (2012). 10.1038/ncomms1791 22569373

[b14] D’Orsogna M. R. & Rudnick J. Two-level system with a thermally fluctuating transfer matrix element: application to the problem of DNA charge transfer. J. Phys Rev E Stat Nonlin Soft Matter Phys. 66, 041804 (2002). 10.1103/PhysRevE.66.041804 12443225

[b15] Lagerqvist J., Zwolak M. & DiVentra M. Fast DNA sequencing via transverse electronic transport. J. Nano Lett. 6, 779–782 (2006). 10.1021/nl0601076 PMC2556950 16608283

[b16] Lagerqvist J., Zwolak M. & DiVentra M. Influence of the environment and probes on rapid DNA sequencing via transverse electronic transport. Biophys J 93, 2384–2390 (2007). 17526560 10.1529/biophysj.106.102269 PMC1965446

[b17] Xu K. DNA Circuit System and Charge Transfer Mechanism. J. Engineering. 5, 381–385 (2013).

[b18] Zhang X.-G. *et al.* First-Principles Transversal DNA Conductance Deconstructed. J. Biophys. 91, 4–6 (2006). 10.1529/biophysj.106.085548 PMC1479083 16679371

[b19] Zikic R. *et al.* Characterization of the tunneling conductance across DNA bases. Phys. Rev. E 74, 011910–011919 (2006). 10.1103/PhysRevE.74.011919 16907139

[b20] MECA Electronics, INC. *Adaptors*. Available at: http://www.e-meca.com/cables-and-adapters/bulkhead-adapters (Accessed: 25th May 2016).

[b21] Gamry. Potentiostat. Available at: http://www.gamry.com (Accessed: 25th May 2016).

[b22] Kasap S. O. Principles of Electronic Materials and Devices (2nd ed) (Mc Graw Hill, New York, 2002).

[b23] Sze S. M. & Ng K. Physics of Semiconductor Devices (ed. 3) (John Wiley & Sons, Inc., Hoboken, New Jersey, 2007).

[b24] Cutnell J. D. & Johnson K. W. Physics (ed. 9) (John Wiley & Sons, Inc., New Jersey, 2012).

